# Development and effectiveness of a BOPPPS teaching model-based workshop for community pharmacists training

**DOI:** 10.1186/s12909-024-05282-9

**Published:** 2024-03-15

**Authors:** Bingzheng Shen, Yating Chen, Yue Wu, Yu Lan, Xiaoqin He, Na Wang, Jun Liu, Yan Yu

**Affiliations:** 1 Department of Pharmacy, Renmin Hospital, Wuhan University, Wuhan, China; 2 Department of Clinical Pharmacy, School of Pharmaceutical Science, Wuhan University, Wuhan, China; 3 Teaching Affair Office, The First Clinical College, Wuhan University, Wuhan, China; 4 Health Service Center, Xianghe Community, Wuhan, China; 5 Department of Gastroenterology, Tongji Hospital of Tongji Medical College, Huazhong University of Science and Technology, Wuhan, China

**Keywords:** BOPPPS teaching model, Workshop strategy, Continuing education training, Outcome, Community pharmacists

## Abstract

**Background/Aim:**

With the pharmaceutical innovation and clinical knowledge updating, the continuing education and on-the-job training are extremely important for improving community pharmacists’ professional competence. Previous training often adopted traditional lecture-based teaching, and the efficacy was limited. The aim of this study is to develop a new strategy for community pharmacist training.

**Methods:**

Based on the BOPPPS (Bridge-in, Objective, Pre-assessment, Participatory Learning, Post-assessment and Summary) teaching model and workshop method, a continuing on-the-job training program was constructed. Participates were randomly and evenly divided into two groups by random number table method. Twenty-four community pharmacists in total completed all training contents and evaluation components in this study. Twelve pharmacists in experimental group were trained via this new BOPPPS-based workshop, while others still adopted traditional didactic lecture-based approaches.

**Results:**

After training, quantitative examination combined with clinical pharmacy practice tests were carried out to evaluate the effectiveness and outcomes of two training groups. For written exam, the total scores from the BOPPPS-based workshop group (82.67 ± 4.70) was higher than that of traditional lectured-base group (73.75 ± 6.15) (*P* < 0.001). Encouragingly, compared with the results of practical ability assessment from traditional training group (71.75 ± 4.75), the pharmacists receiving BOPPPS-based workshop training presented more excellent performance (78.25 ± 5.03), which displayed statistically significant differences (*P* < 0.01). In addition, an anonymous questionnaire was used to survey trainees’ feelings after completing this continuing education program. The results revealed that the BOPPPS-based workshop can bring a better learning experience than traditional lecture-based training, and the percentages of positive response to each item were more than 91.7%.

**Conclusions:**

Through multi-dimensional evaluation, it was suggested that our BOPPPS-based workshop achieved desired training effects. Moreover, our research also demonstrated that this strategy had advantages of stimulating inspiration, autonomous learning, team-work spirit and pharmacy practice improvement. It may provide a reference of innovative training method for community pharmacists.

**Supplementary Information:**

The online version contains supplementary material available at 10.1186/s12909-024-05282-9.

## Introduction

Community health service center is one of the important public institution for residents’ health. In China, The primary health care system was initially established in the 1980s [1]. But little attention was paid to training for professional and technical personnel. Different from health workers in comprehensive teaching hospital, community pharmacist who lack efficient and systematic training can not fully meet the needs for health services. More seriously, the neglect of continuing education furtherly caused knowledge obsolescence and lack of clinical thinking. After suffering from the COVID-19 epidemic, the key role of community health care services has been fully reflected in public health system. Recently, Chinese National Health Commission has been trying to strengthen the primary health care system so as to provide better health care services [2].

The best way to change current state is to develop an on-the-job training program for community health professionals. This training program is an important part of the continuing education for community pharmacists in Xianghe Community, who are required to get credits of professional study per year (one credit for every three class hours of learning). A total of thirty-four pharmacists serve the community. Except for those who were on leave due to illness or personal issues, all of them should be invited to take part in this program. The core topic of this training program mainly focused on the rational use of medications and clinical practice skills for community pharmacists. The purpose of this study is to establish a continuing education course and feasible training system for community pharmacist. The BOPPPS model, composed of six important sections (Bridge-in, Objective, Pre-assessment, Participant learning, Post-assessment and Summary), is an effective teaching model and firstly proposed by Douglas Kerr from University of British Columbia in Canada [3]. It It has been widely used for medical undergraduate teaching and clinical internship [4–6], but rarely applied in pharmacists’ continuing training. For trainees, workshop learning may create new ideas, inspire them to further exploration, provide opportunities for networking, promote cooperation and having fun while learning [7–9].

Hence, combined the BOPPPS teaching model with workshop learning, we developed a new training program for community pharmacists, including both professional knowledge and pharmaceutical care skills. The ultimate aim is to enhance their professional competence and provide excellent pharmaceutical services for community residents. We hope to provide a reference for the construction and implementation of continuing education training for community pharmacists.

## Methods

### Participants

All people who registered for annual continuing education program were included as possible candidates. In this study, the inclusion criteria were: (1) community pharmacist in primary health care center or institute, (2) currently working on the front line, (3) more than one year of community pharmacy service experience, (4) the ability to use smart phone or computer. Participants were randomly divided into either a BOPPPS model-based workshop or a traditional lecture-based classroom learning. The training time of both BOPPPS-based workshop and traditional lecture-based teaching was the same. The total duration of training was twelve class hours, and one class period lasted forty-five minutes. Each section of training contents took three class hours with two ten-minute short breaks. This study was approved by the Research Ethics Board of the Health Service Center of Xianghe Community.

### BOPPPS model-based workshop

**Fig. 1:**
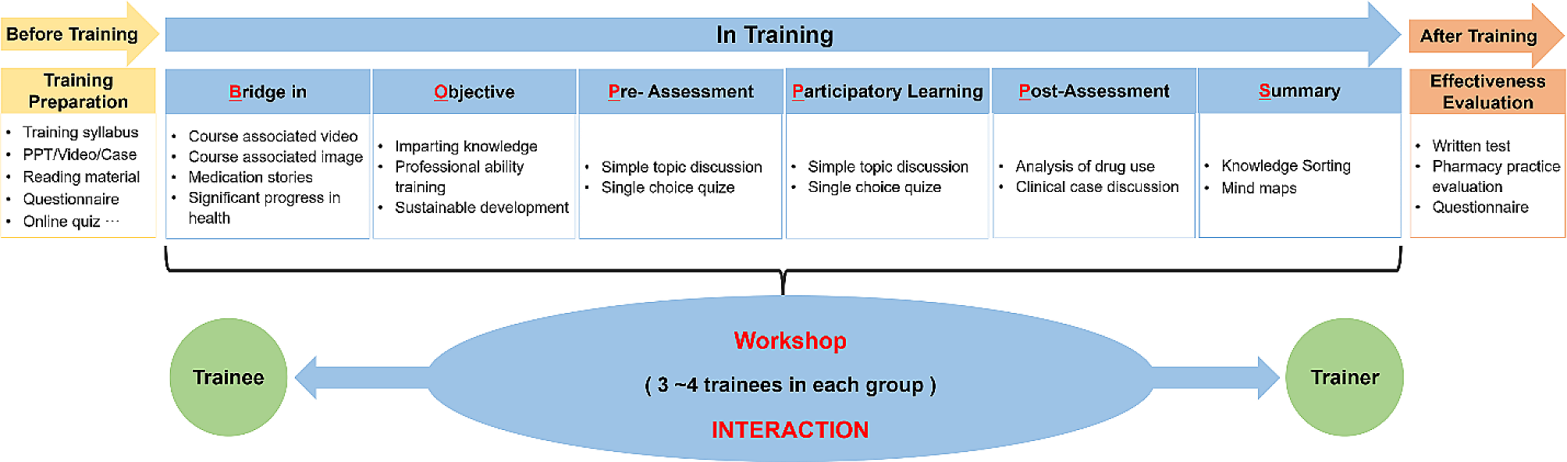
Overall design of BOPPPS model-based workshop training for community pharmacists

The BOPPPS effective teaching model was used for the design and development of this new training program [6, 10–12]. As shown in Fig. 1, it was composed of six modules. Participants were encouraged to form a few small workshop groups, and each group was consisted of three to five trainees. Before training, video and image was used as bridge to attract their interests. The objective module can make trainees know the purpose of learning. The pre-assessment, including single best answer question, allowed the instructor to better understand trainees’ professional level. Participatory learning was the main body of our program, and all learners were involved as actively as possible in the training process. In addition, workshop required pharmacists to explore pharmacy service cases using the pharmaceutical skill they learned, which can also provide diverse insights into training topics and motivate trainees to explore new field of interest. Post-assessment section allowed the trainer to evaluate learners’ understanding of the content and directly reflected the outcomes of this training. The summary part signaled the end of each training unit. The instructors and trainees were given the opportunities to summarize the teaching content and conclude their learning experiences, respectively. The specific training contents were shown in Table 1.

**Table 1:** The training contents of BOPPPS model-based workshop

Sections (Training contents)	Materials for training			Time allocation for workshop training / 90 min			
	images	Videos	Cases	Theoretical teaching/min	Intra-group discussion/min	Presentation/min (three groups)	Summery/min
**Section 1: Prescription checking** • Outpatient prescription checking • Inpatient prescription checking • Narcotic drug prescription checking	4	2	2	30	10	15 × 3 = 45	5
**Section 2: Rational use of drugs for chronic diseases** • Rational use of drugs for Cardiovascular and cerebrovascular diseases • Rational use of drugs for cancer • Rational use of drugs for chronic respiratory diseases	5	4	3	30	10	15 × 3 = 45	5
**Section 3: Rational use of drugs for special patient populations** • Rational use of drugs for elderly patients • Rational use of drugs for infants and children • Rational use of drugs for pregnant women	4	3	3	40	15	10 × 3 = 30	5
**Section 4: First aid skills** • Cardiopulmonary resuscitation • Heimlich manoeuvre • Bandaging and hemostasis • External fixation with splints	12	6	4	40	15	10 × 3 = 30	5

### Traditional lecture-based training

Participants in the traditional group received lecture-based training courses. This traditional didactic training approach had historically been the primary on-the-job training modality in continuing education for community health workers. At its core, it has been a trainer-centered style that relies on the one-way passive transfer of knowledge from the instructor to the trainees. The teaching contents in traditional training group also covered all key teaching points as the same as the BOPPPS model-based workshop.

### Evaluation of training effects

All participants were required to provide basic personal information via Wenjuanxing APP. By the end of this training, every trainee need to take comprehensive exams including written test and pharmaceutical care assessment. The written test was composed of single-answer question, multiple-answers question and prescription analysis. On the other hand, the pharmaceutical care ability was assessed through trainees’ participation in pharmacy service work, where pharmacist was mainly focused on the rational use of medications in patients. It was comprised of three parts, involving medication guidance to outpatients, rational use of medication for inpatients and drug treatment recommendations in clinical case discussion. The type, quantity and score of written test and pharmacy practice assessment in comprehensive evaluation were exhibited in Supplementary Table S1.

Besides the results of comprehensive tests, the trainees’ real feelings of learning was also considered as an important aspect to reflect the quality and effect of this training program. In consequence, an anonymous survey was performed, and each item in the questionnaire was a single best answer question. It can promote participants completed all survey contents timely and smoothly. The detail of each question was exhibited in Table 2.

**Table 2:** The questionnaire of trainees’ experience for all participants

Survey item	Agree	Neutral	Disagree
1. Training contents can keep pace with the times.	□	□	□
2. The training contents can guide the clinical pharmacy practice.	□	□	□
3. It is easy to understand the knowledge points.	□	□	□
4. The effective communication between trainers and learners is very smooth.	□	□	□
5. The training atmosphere is active, and the training program is interactive.	□	□	□
6. The case selection for the training is reasonable.	□	□	□
7. Trainees are highly engaged in the training process.	□	□	□
8. This training can improve our pharmacy skills and professional competence.	□	□	□
9. This training achieves my expectations.	□	□	□
10. Such on-the-job training for community pharmacists needs to be ongoing.	□	□	□

### Statistical analysis and data management

The software of GraphPad Prism version 9.0 was used for the descriptive and primary statistic analyses. The measured data were shown as means ± SD for continuous variable. After checking for normal distribution of the corresponding data, statistical parameters were calculated to analyze the relationships and difference between variables in different training groups. A *P*-value less than 0.05 could be considered statistically significant. After community pharmacists successfully enrolled in our training project, an unique and independent trainee code was assigned. To protect their privacy and avoid subjective bias, every pharmacist was identified only by their ID numbers. All learners’ personal information and training data were managed through the strategy of two people with two different passwords.

## Results

### Characteristics of trainees

**Fig. 2:**
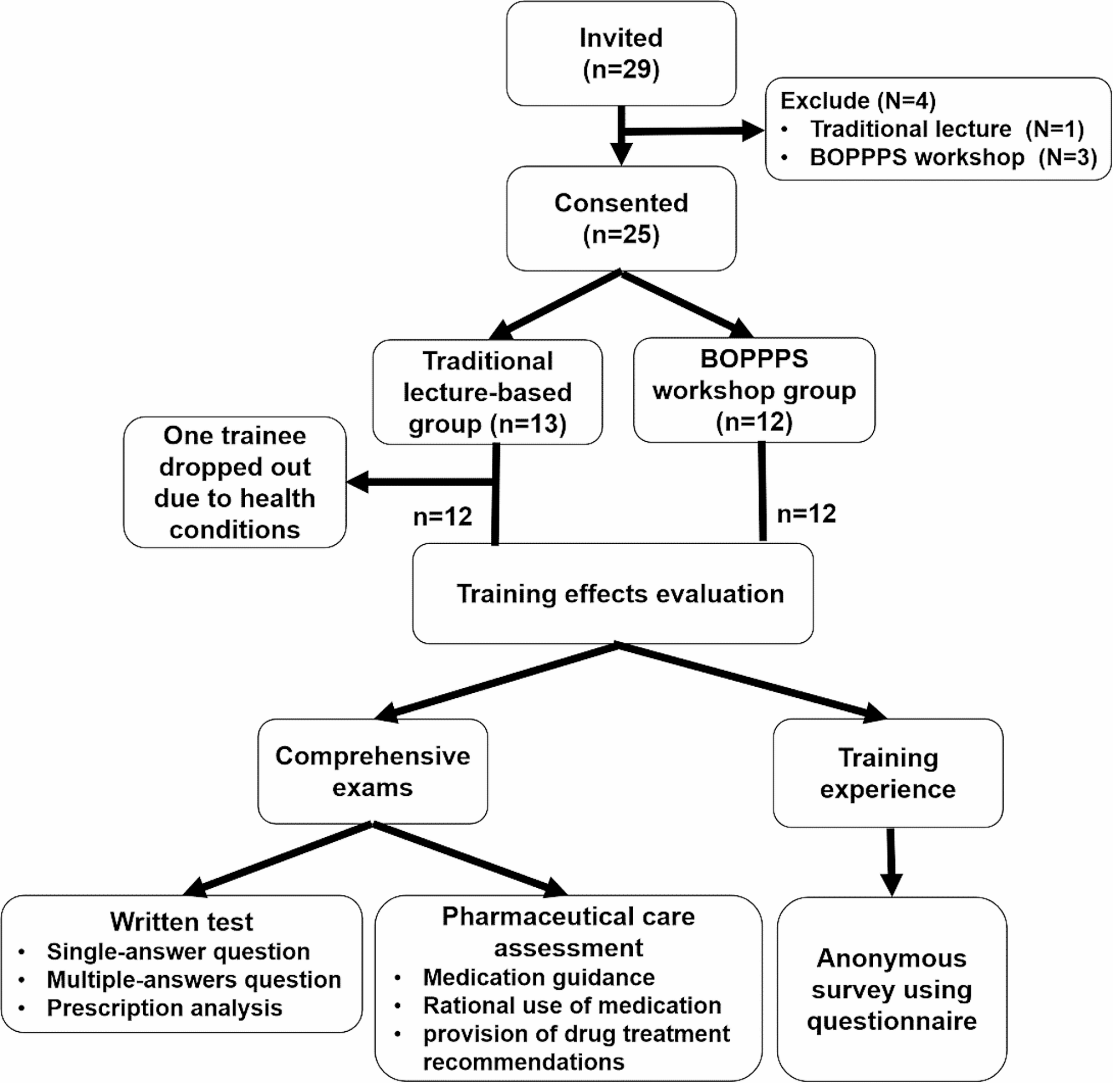
Overview of research design and training process

The overview of our research design and training process were shown in Fig. 2. A total of the thirty-one community pharmacists were invited to this continuing training, and twenty-nine consented to take part in this research. After screening, four people did not meet the inclusion criteria. Of the twenty-five participants, twelve learners was randomized to the BOPPPS-based workshop group, while a group of thirteen were randomized to the traditional lecture-based training. Due to the health status, one pharmacist in traditional training group didn’t complete all required training contents. Consequently, based on the twenty-four learners remaining, the data were collected, calculated and analyzed for our research. The overall completion rates for BOPPPS workshop group and traditional lecture-based group were 100% and 92.3%, respectively. The basic socio-demographic data for the trainees were shown in Table 3. After the COVID-19 epidemic, the investment and construction for primary medical institutions had been greatly improved. More to the point, many young and highly educated pharmaceutical talents had served as community health workers. Nearly half of the participants possessed a master’s degree, and more than 80% of them had middle professional title of pharmacist-in-charge or above. About four-fifths of trainees were women, which the ratio of female participants was in accordance with the data of the global human resources for health workforce [13–14].

**Table 3:** Social-demographic profile of all twenty-four trainees in two study groups

Characteristic	Category	Traditional lecture-based group (*N* = 12)	BOPPPS-based workshop group (*N* = 12)
Gender	Male	2 (16.7)	2 (16.7)
	Female	10 (83.3)	10 (83.3)
Age	21 ~ 30	6 (50.0)	6 (50.0)
	31 ~ 40	2 (16.7)	3 (25.0)
	41 ~ 50	3 (25.0)	2 (16.7)
	51 ~ 60	1 (8.3)	1 (8.3)
Education	Junior college	2 (16.7)	2 (16.7)
	Bachelor	4 (33.3)	5 (41.7)
	Master	6 (50.0)	5 (41.7)
	Doctor	0 (0.0)	0 (0.0)
Professional title	Pharmacist	4 (33.3)	5 (41.7)
	Pharmacist-in-charge	5 (41.7)	5 (41.7)
	Associate chief pharmacist	2 (16.7)	1 (8.3)
	Chief pharmacist	1 (8.3)	1 (8.3)
Years of working	0 ~ 10	8 (66.7)	9 (75.0)
	11 ~ 20	3 (25.0)	2 (16.7)
	20 ~ 30	1 (8.3)	1 (8.3)
	> 30	0 (0.0)	0 (0.0)

### The outcome of written-test and pharmacy practice ability

**Fig. 3:**
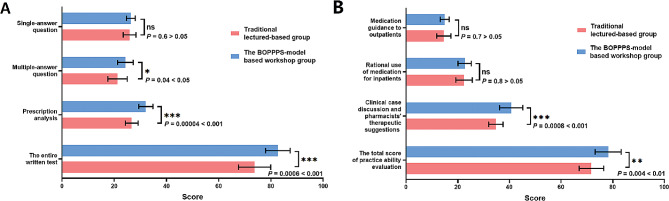
Comprehensive evaluation of outcomes between two training groups. (**A**) Comparison of total score and score of each type question from written-tests. (**B**) Comparison of total score and score of each pharmacy-practice topic from clinical pharmacy practice evaluation

The results of quantitative evaluation, including all learners’ final tests scores and assessment of pharmacy service capacity, were collected to explore and compare training outcomes in different groups. In total, the twenty-four community pharmacists finished all required items were involved and assessed. The results were exhibited in Fig. 3A. The total scores of written-tests from BOPPPS workshop and traditional training were 82.67 ± 4.70 and 73.75 ± 6.15, respectively. It showed a striking difference with *p* < 0.001. The comparison of three types of questions in written exam between two training groups was helpful to analyze the reasons for the differences. The scores of single-answer question reflecting the mastery of basic concepts showed no difference between two groups (*p* > 0.05). But the results of multiple-answers question and prescription analysis used for assessing comprehensive understanding and application, displayed statistical differences, which the *p* values were 0.04 (*p* < 0.05) and 0.0004 (*p* < 0.001), respectively.

Different from traditional test after training, the practice ability evaluation can better embody the effectiveness of this BOPPPS-based workshop on improving professional competency (Fig. 3B). After training, the total scores of workshop and traditional groups were 71.75 ± 4.75 and 78.25 ± 5.03, and the differences were statistically significant (*p* < 0.01). Analysis of trainees’ performance in each specific assessment revealed that there was no difference in medication guidance to outpatients and rational use of medication for inpatients (*p* > 0.05). The reason why learners from BOPPPS-based workshop group achieved high scores was that they displayed much more excellent performance in clinical case discussion and pharmacists’ therapeutic suggestions (*p* < 0.001).

### Questionnaire

**Fig. 4:**
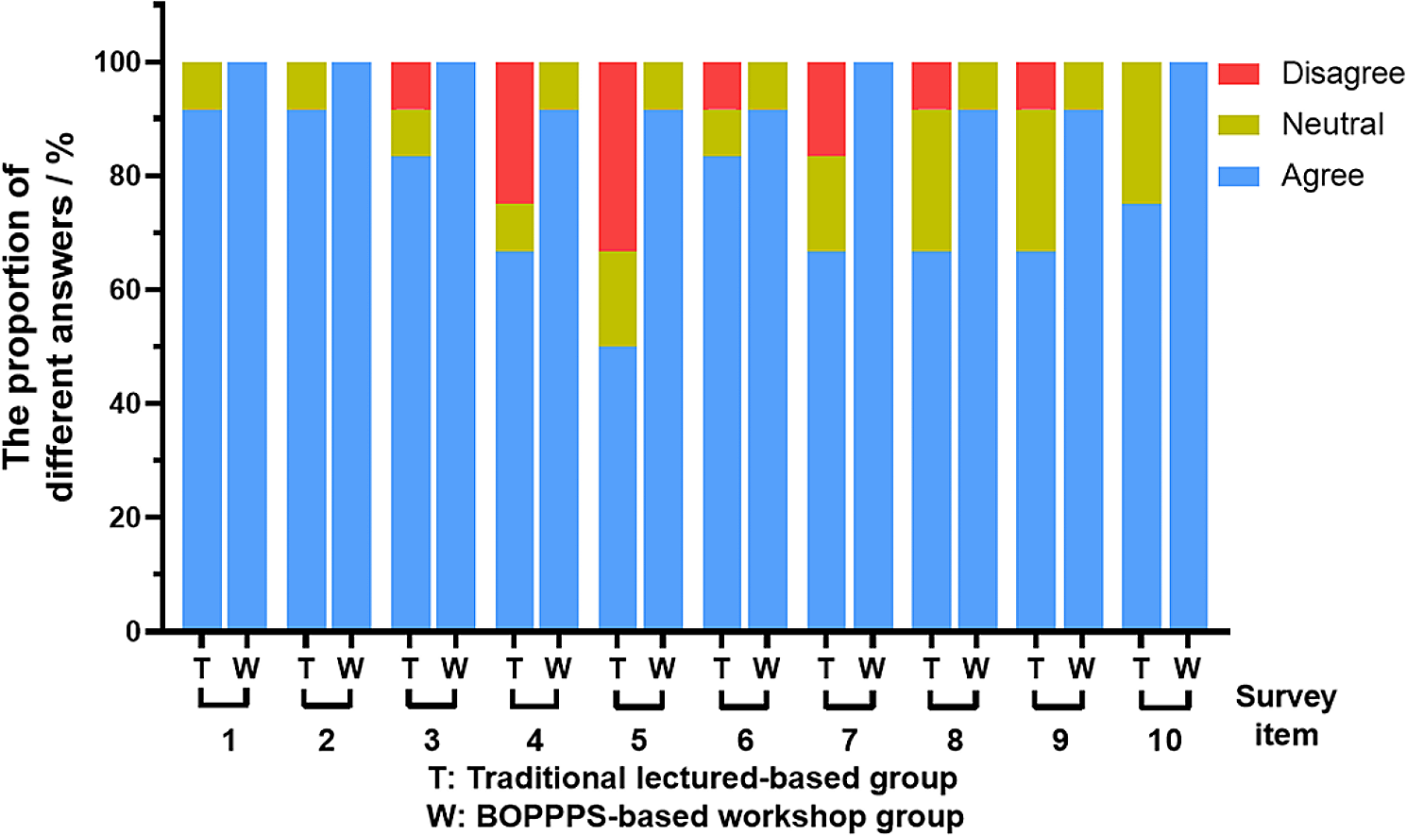
The percentage of each option for every item from questionnaire in two research groups

The questionnaire survey was carried out to obtain and explore learners’ attitudes and feelings on their training experiences and satisfaction degree via on-line Wenjuanxing App. The survey item in e-questionnaire mainly focused on the the training content, practicability, interactivity and professional competence and potential stimulation. For trainees, this anonymous survey was not mandatory, and it wound not have any impact on their assessment results. Fortunately, all twenty-four learners who had received the training program completely finished and returned the e-questionnaire. The results of survey analysis for trainees’ educational experience were displayed in Fig. 4. In workshop group, more than 90% of trainees chose positive comments on each question, and none of the trainees selected the negative option. Nevertheless, training feelings from lecture-based group was not very satisfying. The percentage of positive options ranged from 60 to 80%, four items even presented negative options with the proportion from 10 to 20%. It was indicated that the BOPPPS-based workshop could provide better learning experience, so as to achieve satisfactory outcomes.

## Discussion

In China, community pharmacists play an irreplaceable role in the primary health-care system. They specialize in the preparation, dispensing, clinical medication managements, and also provide pharmaceutical guidance and drug therapy recommendations [15–17]. After 3 years of the COVID-19 pandemic, the hardware construction and upgrading in community health service centers were basically completed. However, more important than instrument and equipment is the continuous improvement of health workers’ theoretical knowledge and professional skill. The primary aim of continuing education is to deepen the basic knowledge, learn about the latest pharmacy progress and cultivate the clinical thinking so that community pharmacists can apply professional skills they learned to solve the medication-related problems encountered in clinical health care. Many evidences in education research have questioned the efficacy of traditional lecture-based learning [18–20], which has led us to develop an alternative strategy and tactic of training for community pharmacist. The effective training model named BOPPPS was adopted to build the overall framework and arrange the content of this training. It emphasized that the educational program should focus on the outcome and sustainable career development.

Participation, acceptance and learning experiences are the three key factors determining the effectiveness of this training programs for community pharmacists. Although the BOPPPS model can optimize the organization of training contents and be of great help to enhance training quality, many studies have found that it can achieve the much better teaching effects when combined with other methods [21–23]. Consequently, workshop strategy was used, and the BOPPPS based-workshop can fit well with the needs of continuing medical education and align well with interactive training principles. Workshop can be an effective and powerful medium bridging the training instructors and learners together for potentiating a deeper learning, which are great for creating the learning atmosphere of face-to-face discussion. We prefer the training method of workshop for community pharmacist lies in its advantages of knowledge updating, professional skill construction, problem-solving and self-improvement. In addition to continuing medical training, it is also used for undergraduate and postgraduate education [24–26].

All trainees from both BOPPPS-based workshop and traditional group had the same training objectives and learning materials. The key difference between these two groups lied in the organization and implementation of training classes. By comparing the two groups adopting different training methods, our research was carried out to explore and analyze whether the BOPPPS-based workshop could better improve professional level and achieve expected training outcomes. Our findings of this study were as follows: (1) For the specialized knowledge learners need to be memorized, there was no difference between the two training groups; (2) Trainees from workshop group were fairly good at using theories knowledge to solve problems in clinical pharmacy practice; (3) The BOPPPS-based workshop program possessed the advantages of better engagement, interactivity and training effectiveness; (4) For the participates, this continuing training curriculum integrated BOPPPS model and workshop method can achieve higher acceptance and satisfaction.

In the adult learning process, they have a limited attention span of up to 20 min or so [27–29]. Therefore, workshops with BOPPPS model was designed to properly allocate time for theory lecture and inter-group discussion. And the teaching material should be cutting-edge, contextual and practical, which is conducive to trainees’ interaction and discussion-based learning. Another key point is that the learning activities need to be aligned with the learning contents and objectives during the process of BOPPPS workshop training. Trainer need to be able to take control of the class and organize the didactical activities around the topic.

This BOPPPS workshop strategy can help trainees to know training objects easily, enter the active state quickly, develop problem-solving skills and foster critical thinking, but its main disadvantage is that it need take some extra time for older trainees to adapt to this new learning rhythm, which may affect teaching effectiveness at the beginning of training. In recent ten years, with the emphasis on teaching research and reform, many new teaching methods have been gradually applied to undergraduate and postgraduate education. Most trainees aged 21–30 and some of them aged 31–40 may learn professional curriculum through innovative educational strategies. Therefore, it was estimated that more than half of the community pharmacists involved in this research have the experience with active learning methods. Therefore, with the passage of time, the new training strategy will be accepted and well liked by more and more trainees.

Trainees’ questionnaire displayed that the advantages of BOPPPS-based workshop involve the convenience to immediately utilize theoretical knowledge in practical case discussion and effective improvement of training experience. In addition, the efficient interactions between the instructors and learners were also attributable to the better outcomes of this new training method.

## Limitations

However, due to the limitations, the results of this research need to be interpreted with caution. For one thing, only a small number of community pharmacists included in our research and thus might not be totally reflective of the perception of all community pharmacists in the whole country. For another, the questionnaire survey did not involve quantitative research, which the main purpose was to understand the acceptability and perception of all participants.

## Conclusion

Compared with the traditional lecture-based on-the-job education, this new training strategy displayed obvious advantages. The results of quantitative written and pharmacy service tests as well as anonymous questionnaire manifested that this new training program exhibited an effective way to receive latest professional knowledge and improve clinical practice ability. More important, multiple ways of clinical thinking and reasoning ability they learned from BOPPPS workshop might be very helpful for their sustainable career development. The workshop method with BOPPPS teaching model can stimulate pharmacist’s learning interest and initiative, enhance their ability to solve practical problems in clinical pharmacy practice. Therefore, the BOPPPS-model based workshop we developed is worth applying in on-the-job continuing education for community pharmacists.

### Electronic supplementary material

Below is the link to the electronic supplementary material.

Supplementary Material 1

## Data Availability

The data displayed in our research are available on request from the corresponding author.

## References

[1] Zheng Q, Shi L, Pang T, Leung W (2021). Utilization of community health care centers and family doctor contracts services among community residents: a community-based analysis in Shenzhen, China. BMC Fam Pract.

[2] Xiong S, Cai C, Jiang W, Ye P, Ma Y, Liu H, Li B, Zhang X, Wei T, Sun H, Hone T, Peiris D, Mao L, Tian M (2022). Primary health care system responses to non-communicable disease prevention and control: a scoping review of national policies in Mainland China since the 2009 health reform. Lancet Reg Health West Pac.

[3] Sibley J, Canuto L. Guide to Teaching for New Faculty at UBC; The University of British Columbia: Vancouver, BC, Canada, 2010.

[4] Chen L, Tang XJ, Chen XK, Ke N, Liu Q (2022). Effect of the BOPPPS model combined with case-based learning versus lecture-based learning on ophthalmology education for five-year paediatric undergraduates in Southwest China. BMC Med Educ.

[5] Li Z, Cai X, Zhou K, Qin J, Zhang J, Yang Q, Yan F (2023). Effects of BOPPPS combined with TBL in surgical nursing for nursing undergraduates: a mixed-method study. BMC Nurs.

[6] Xu Z, Che X, Yang X, Wang X (2023). Application of the hybrid BOPPPS teaching model in clinical internships in gynecology. BMC Med Educ.

[7] Seymour NE, Paige JT, Arora S, Fernandez GL, Aggarwal R, Tsuda ST, Powers KA, Langlois G, Stefanidis D (2016). Putting the MeaT into TeaM training: Development, Delivery, and evaluation of a Surgical Team-Training Workshop. J Surg Educ.

[8] Simmons L, Leavitt L, Ray A, Fosburgh B, Sepucha K (2016). Shared decision making in common chronic conditions: impact of a Resident Training Workshop. Teach Learn Med.

[9] Lai NM, Ngim CF, Fullerton PD (2012). Teaching medical students neonatal resuscitation: knowledge gained and retained from a brief simulation-based training workshop. Educ Health (Abingdon).

[10] Li P, Lan X, Ren L, Xie X, Xie H, Liu S (2023). Research and practice of the BOPPPS teaching model based on the OBE concept in clinical basic laboratory experiment teaching. BMC Med Educ.

[11] Ma X, Ma X, Li L, Luo X, Zhang H, Liu Y (2021). Effect of blended learning with BOPPPS model on Chinese student outcomes and perceptions in an introduction course of health services management. Adv Physiol Educ.

[12] Yang Y, You J, Wu J, Hu C, Shao L (2019). The Effect of Microteaching combined with the BOPPPS Model on Dental materials Education for Predoctoral Dental Students. J Dent Educ.

[13] Grant VJ, Robinson E, Muir P (2004). Sex ratios in healthcare occupations: population based study. BMJ.

[14] Boniol M, McIsaac M, Xu L, Wuliji T, Diallo K, Campbell J. Gender Equity in the Health Workforce: Analysis of 104 Countries. World Health Organization. 2019, Available online at: https://apps.who.int/iris/bitstream/handle/10665/311314/WHO-HIS-HWF-Gender-WP1-2019.1-eng.pdf (Accessed December 14, 2023).

[15] Al-Taani GM, Ayoub NM (2022). Community pharmacists’ routine provision of drug-related problem-reduction services. PLoS ONE.

[16] Ayele AA, Islam MS, Cosh S, East L (2021). Involvement and practice of community pharmacists in maternal and child health services: a systematic review. Res Social Adm Pharm.

[17] Mallinder A, Martini N (2022). Exploring community pharmacists’ clinical decision-making using think aloud and protocol analysis. Res Social Adm Pharm.

[18] Chotiyarnwong P, Boonnasa W, Chotiyarnwong C, Unnanuntana A (2021). Video-based learning versus traditional lecture-based learning for osteoporosis education: a randomized controlled trial. Aging Clin Exp Res.

[19] Alhazmi A, Quadri MFA (2020). Comparing case-based and lecture-based learning strategies for orthodontic case diagnosis: a randomized controlled trial. J Dent Educ.

[20] Li T, Wang W, Li Z, Wang H, Liu X (2022). Problem-based or lecture-based learning, old topic in the new field: a meta-analysis on the effects of PBL teaching method in Chinese standardized residency training. BMC Med Educ.

[21] Wang S, Xu X, Li F, Fan H, Zhao E, Bai J (2021). Effects of modified BOPPPS-based SPOC and flipped class on 5th-year undergraduate oral histopathology learning in China during COVID-19. BMC Med Educ.

[22] Li S, Liu Q, Guo S, Li Y, Chen F, Wang C, Wang M, Liu J, Liu X, Wang D, Li E (2023). Research on the application of the blended BOPPPS based on an online and offline mixed teaching model in the course of fermentation engineering in applied universities. Biochem Mol Biol Educ.

[23] Liu XY, Lu C, Zhu H, Wang X, Jia S, Zhang Y, Wen H, Wang YF (2022). Assessment of the effectiveness of BOPPPS-based hybrid teaching model in physiology education. BMC Med Educ.

[24] Zhang Q, Zeng T, Chen Y, Li X (2012). Assisting undergraduate nursing students to learn evidence-based practice through self-directed learning and workshop strategies during clinical practicum. Nurse Educ Today.

[25] Roden JA, Jakob S, Roehrig C, Brenner TJ (2018). Preparing graduate student teaching assistants in the sciences: an intensive workshop focused on active learning. Biochem Mol Biol Educ.

[26] Fatumo S, Shome S, Macintyre G (2014). Workshops: a great way to enhance and supplement a degree. PLoS Comput Biol.

[27] Thiessen ED, Girard S, Erickson LC (2016). Statistical learning and the critical period: how a continuous learning mechanism can give rise to discontinuous learning. Wiley Interdiscip Rev Cogn Sci.

[28] Murphy M (2008). Matching workplace training to adult attention span to improve learner reaction, learning score and retention. J Instruction Delivery Syst.

[29] Belay HT, Ruairc Ó, Guérandel B (2019). Workshops: an important element in medical education. BJPsych Adv.

