# Supplementary material for: Development and effectiveness of a BOPPPS teaching model-based workshop for community pharmacists training
Source: BMC Med Educ. 2024 Mar 15;24:293. doi: 10.1186/s12909-024-05282-9 (PMC10943807; doi:10.1186/s12909-024-05282-9)
Supplement: Supplementary file 1 — Supplementary Material 1 [file 12909_2024_5282_MOESM1_ESM.docx]

**Supplementary Table S1**

**Table S1.** The type, quantity and score of written test and pharmacy practice assessment in comprehensive evaluation

| **Comprehensive evaluation** | **Type** | **Quantity** | **Score** |
| --- | --- | --- | --- |
| Written test  (100 points in total) | Single-answer question | 15 | 30 |
|  | Multiple-answers question | 10 | 30 |
|  | prescription analysis | 2 | 40 |
| Pharmacy practice assessment  (100 points in total) | Medication guidance to outpatients | 2 | 20 |
|  | Rational drug use for inpatients | 2 | 30 |
|  | Drug treatment recommendations  in clinical case discussion | 2 | 50 |
